# Effect of Single and Combined Monochromatic Light on the Human Pupillary Light Response

**DOI:** 10.3389/fneur.2018.01019

**Published:** 2018-11-29

**Authors:** Maria A. Bonmati-Carrion, Konstanze Hild, Cheryl M. Isherwood, Stephen J. Sweeney, Victoria L. Revell, Juan A. Madrid, Maria A. Rol, Debra J. Skene

**Affiliations:** ^1^Chronobiology Laboratory, Department of Physiology, IMIB-Arrixaca, University of Murcia, Murcia, Spain; ^2^Ciber Fragilidad y Envejecimiento Saludable, Madrid, Spain; ^3^Advanced Technology Institute and Department of Physics, University of Surrey, Guildford, United Kingdom; ^4^Chronobiology, Faculty of Health and Medical Sciences, University of Surrey, Guildford, United Kingdom; ^5^Surrey Clinical Research Centre, Faculty of Health and Medical Sciences, University of Surrey, Guildford, United Kingdom

**Keywords:** pupillometry, light, pupillary light reflex, ipRGC, melanopsin, human melanopic lux

## Abstract

The pupillary light reflex (PLR) is a neurological reflex driven by rods, cones, and melanopsin-containing retinal ganglion cells. Our aim was to achieve a more precise picture of the effects of 5-min duration monochromatic light stimuli, alone or in combination, on the human PLR, to determine its spectral sensitivity and to assess the importance of photon flux. Using pupillometry, the PLR was assessed in 13 participants (6 women) aged 27.2 ± 5.41 years (mean ± SD) during 5-min light stimuli of purple (437 nm), blue (479 nm), red (627 nm), and combinations of red+purple or red+blue light. In addition, nine 5-min, photon-matched light stimuli, ranging in 10 nm increments peaking between 420 and 500 nm were tested in 15 participants (8 women) aged 25.7 ± 8.90 years. Maximum pupil constriction, time to achieve this, constriction velocity, area under the curve (AUC) at short (0–60 s), and longer duration (240–300 s) light exposures, and 6-s post-illumination pupillary response (6-s PIPR) were assessed. Photoreceptor activation was estimated by mathematical modeling. The velocity of constriction was significantly faster with blue monochromatic light than with red or purple light. Within the blue light spectrum (between 420 and 500 nm), the velocity of constriction was significantly faster with the 480 nm light stimulus, while the slowest pupil constriction was observed with 430 nm light. Maximum pupil constriction was achieved with 470 nm light, and the greatest AUC_0−60_ and AUC_240−300_ was observed with 490 and 460 nm light, respectively. The 6-s PIPR was maximum after 490 nm light stimulus. Both the transient (AUC_0−60_) and sustained (AUC_240−300_) response was significantly correlated with melanopic activation. Higher photon fluxes for both purple and blue light produced greater amplitude sustained pupillary constriction. The findings confirm human PLR dependence on wavelength, monochromatic or bichromatic light and photon flux under 5-min duration light stimuli. Since the most rapid and high amplitude PLR occurred within the 460–490 nm light range (alone or combined), our results suggest that color discrimination should be studied under total or partial substitution of this blue light range (460–490 nm) by shorter wavelengths (~440 nm). Thus for nocturnal lighting, replacement of blue light with purple light might be a plausible solution to preserve color discrimination while minimizing melanopic activation.

## Introduction

The pupillary light reflex (PLR) is a neurological reflex characterized by a reduction in pupil diameter in response to an increase in retinal illumination, as well as the subsequent redilation of the pupil after light cessation. Its main function is to increase the depth of field and image sharpness in bright light conditions.

Rods and cones were the only known mammal photoreceptors until the discovery of melanopsin (1), a photopigment with a peak of sensitivity (λ_max_) at 480 nm, which is expressed in the intrinsically photosensitive retinal ganglion cells (ipRGCs) (2). These ipRGCs project to the suprachiasmatic nuclei (SCN), the circadian pacemaker, and other non-image forming brain areas, such as the olivary pretectal nucleus (OPN), a control center for the PLR (3–7). Thus, ipRGCs participate in a common pathway for the PLR and other processes such as circadian entrainment, either by themselves (intrinsically) or through their connections with the outer retinal photoreceptors (extrinsically), the most efficient wavelengths (humans, λ_max_ 446–477 nm) to both entrain the circadian timing system and inhibit melatonin synthesis (8, 9) being those closer to the maximal sensitivity for ipRGCs. In addition, a relationship between circadian status and PLR has recently been reported (10), indicating a complex inverse relationship between both systems. Once this common pathway and its interactions are further studied, it may be plausible to assess the effect of different lights on the human circadian system through their effects on the PLR.

The human PLR follows a general dynamic (11–14), that can be affected by the intensity, spectral composition (15, 16) and duration of the light stimulus. When the stimulus starts, the pupil shows a rapid constriction until it reaches a minimum size (maximal constriction amplitude). After this early transient response, a pupillary re-dilatation occurs (escape), reaching a more sustained state of partial pupil constriction, which lasts until the end of the light stimulus (17) as well as after termination (post-illumination pupil response, PIPR) (12). According to some studies in primates and humans, the early transient pupil constriction is predominantly driven by cones, while control of the sustained and persistent PIPR seems to correspond to a melanopsin-mediated intrinsic response (12, 18–20). Recent studies, however, have suggested that the outer retinal photoreceptors could also participate in this sustained (21–23) and post-illumination pupil response (PIPR) (24, 25). Despite some limitations of pupillometry, it is possible to infer rod and cone function and the intrinsic activation of ipRGCs independently by analyzing the transient, sustained, and persistent (or PIPR) pupillary response to light stimuli of different wavelengths, intensities, and durations (17).

Apart from their relative specificity on the PLR dynamics, each human retinal photoreceptor exhibits different wavelength sensitivities, based on their corresponding photopigments: λ_max_ 498 nm for rods, λ_max_ 420 nm for S-cones, λ_max_ 530 nm for M-cones, λ_max_ 559 nm for L-cones (26). The maximum sensitivity for melanopsin-containing ipRGCs has been established at 480 nm (2, 6, 27), although other PLR studies in humans indicate peak sensitivities around 490 nm (28), based on ocular photoresponses. In primates intensity thresholds for each photoreceptor are also different, being higher for ipRGCs (~10–11 log quanta/cm^2^/s) (23, 29) than for the classical photoreceptors [cones 2.30 log quanta/cm^2^/s; rods 1.70 log quanta /cm^2^/s, at the cornea level (30)]. Furthermore, it has been proposed that melanopsin's spatial conformation and thus its wavelength sensitivity can switch back from the M to R state by absorbing longer wavelength photons, so-called melanopsin bistability (31, 32). This has also been associated with the PLR, exhibiting increased pupil constriction when the light stimulus was preceded by longer wavelength light (32). Tristability (with two silent and one signaling state) has also been suggested as a mechanism for ipRGC to integrate both time and wavelength (33). However, not all studies have been able to demonstrate this long wavelength potentiation of blue light responses (34, 35), while some studies have proposed the existence of retinal pigment epithelium (RPE)-derived regeneration in melanopsin (36, 37), which could be interpreted as a complementary mechanism [reviewed in (38)].

The study of possible interactions between two monochromatic wavelengths when administered simultaneously, as well as assessment of PLR sensitivity over a high resolution short wavelength range will help to provide knowledge on the effect of polychromatic lights on the PLR.

The aim of this study was thus to achieve a more precise picture of the effects of 5-min monochromatic light stimuli, alone or in combination [long (red) combined with short (blue and purple) wavelength lights], on the human PLR (including PIPR), to determine its spectral sensitivity and to confirm the importance of photon flux as a determinant of the human PLR.

Based on previous knowledge, we hypothesized that blue or purple light would produce different responses when combined with red light as a result of melanopsin bistability, probably increasing the sustained response (greater amplitude) due to the conformational change of melanopsin by red light. Regarding monochromatic short wavelength light, we expected greater pupillary responses under the 460–490 nm light range, and under higher light intensities.

## Materials and methods

### Participants

This study was approved by the University of Surrey Ethics Committee. Volunteers received appropriate information about the study protocol, signed a written informed consent form (in compliance with the Declaration of Helsinki) before being enrolled and were compensated for their participation.

In both experimental conditions participants were healthy, non-smoking volunteers: 13 (6 women) between 19 and 35 years (27.2 ± 5.41 years, mean ± SD) for Study A, and 15 (8 women) between 19 and 35 years (25.7 ± 8.90 years) for Study B. Data from three participants from Study A were excluded from analysis because of very noisy PLR signals that were not interpretable.

All participants declared no medical or mental health disorders and were not taking any medication that could affect circadian rhythms, according to the general health questionnaires completed during the screening period. None of them were shift workers nor had crossed more than two time zones in the 2 months prior to study admission. They kept regular sleep-wake cycles with no reported sleep disorders (Pittsburgh Sleep Quality Index ≤ 5) (39), and were not extreme morning or evening types (40). A full ophthalmic examination including uncorrected vision, near vision corrected, ophthalmoscopy, pupil reactions, Henson Field Test, refraction, intra-ocular pressure, oculomotor status, stereo acuity, accommodation, and color vision by the Ishihara test, was performed to confirm they all were free from any ocular disorders.

### Pre-study measurements

The protocol used was similar to that previously described (41) with participants maintaining a regular, actigraphically (AWL, Cambridge Neurotechnology, UK) monitored sleep/wake schedule for at least 7 days before and throughout the in-laboratory sessions. For 72 h before and during each laboratory session, participants refrained from caffeinated drinks, alcohol, excessive exercise, bright lights, and non-steroidal anti-inflammatory drug intake.

### In-laboratory protocol

#### Protocol for light stimuli presentation

**Figure 1:**
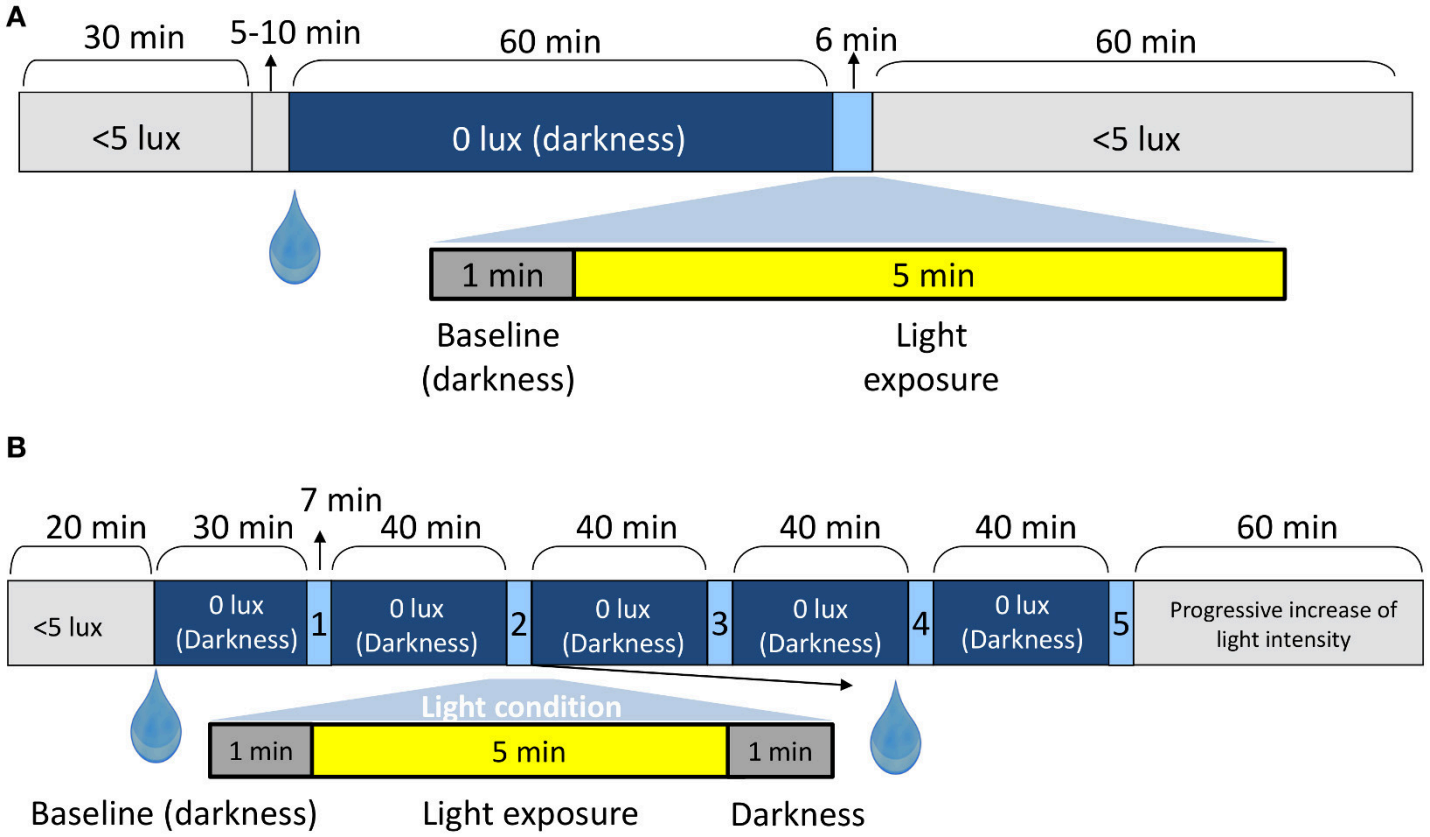
In-laboratory protocol for Study A **(A)** and Study B **(B)**. The drop symbol shows the time when the pupil dilator was administered. The up arrows point to the duration (mins) of the shorter periods.

A randomized, within-subject design was performed. In both experimental conditions A and B, all the sessions were carried out in the morning. In Study A (Figure 1A) the participants (3 per session) arrived at the laboratory and remained seated in dim light (< 5 lux) for 30 min in order to progressively adapt their vision to the dark conditions. Then, he or she received a drop of tropicamide [Minims Tropicamide (1.0%, Chauvin Pharmaceuticals, Romford, UK)] in the right eye to dilate the pupil. After that, the participant remained in darkness (0 lux + eye mask) for an hour prior to pupil recording in order to avoid any confounding effect due to prior light exposures (42). Once this dark adaptation schedule was completed, pupil recording started. For this, the left non-dilated pupil was recorded [220 frames per second] in darkness for 60 s to obtain a baseline, which was later used as a control to normalize the pupil diameter. Then, the light source was turned on and the right eye (pupil dilated) received a light stimulus for 5 min (Figure 1A) while recording the left pupil, thus assessing the consensual reflex. Only one light condition was tested per laboratory session.

The protocol for light stimuli presentation and administration of tropicamide in Study B is shown in Figure 1B and has been detailed in a previous study (10). The pupillary recording followed the same protocol as described for Study A, except that pupil recording continued for 60 s after light offset.

#### Light stimuli characteristics

A 5-min light stimulus was administered to the participant's right eye (dilated) through a specially constructed Ganzfeld sphere (Apollo Lighting, Leeds, UK) coated with white reflectance paint (WRC-680 Labsphere, Pro-Lite Technology, Bedfordshire, UK) to produce patternless illumination. An ultra high-pressure mercury lamp (Focus 100LS3, 100 W, Philips Lighting, Eindhoven, The Netherlands) illuminated the sphere via a fiber optic cable connected to a light box (10, 35, 41)

**Figure 2:**
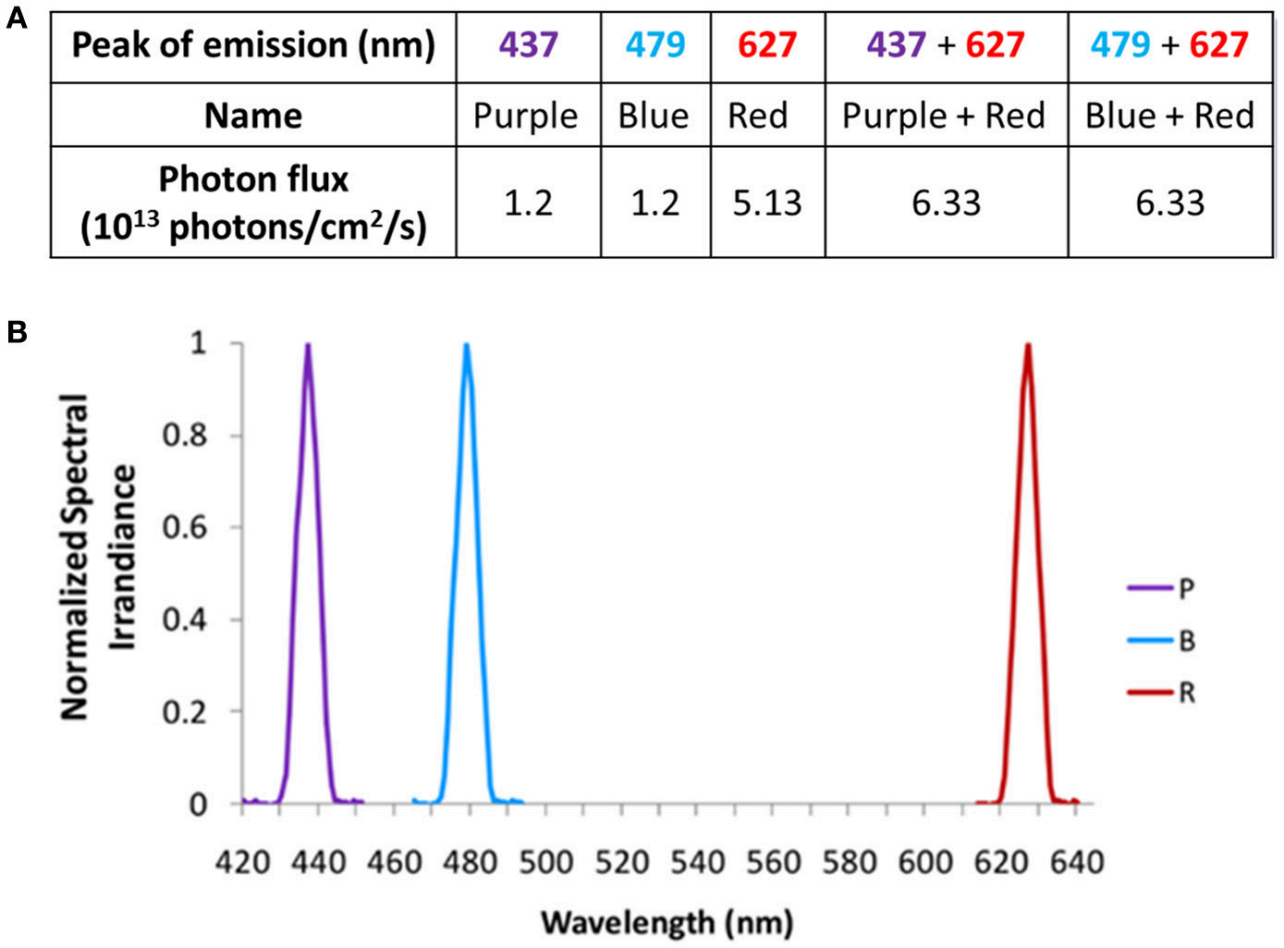
Light wavelengths tested in Study A and their corresponding photon fluxes **(A)**, and normalized spectra **(B)**.

In Study A, monochromatic light (purple, blue, and red) was produced using narrow bandwidth interference filters (Coherent Ealing Europe Ltd., Watford, UK) peaking at 440, 480, and 630 nm (half maximal bandwidth of 10 nm), respectively. The spectra measured at eye level, exhibited peaks at 437, 479, and 627 nm (Figure 2A), respectively, as measured by a calibrated spectrometer (Ocean Optics BV, Dunedin, Florida, USA). Light irradiances were adjusted using neutral density filters (0.10, 0.60, 0.90) (Kodak, Hemel Hempsted, UK) and were verified at the participant's eye level (cornea) using a calibrated radiometer (R203, Macam Photometrics Ltd., Livingston, Scotland) before and after each light exposure.

Purple (437 nm), blue (479 nm), and red (627 nm) monochromatic lights were administered alone or in combination [monochromatic purple + monochromatic red (PR) and monochromatic blue + monochromatic red (BR)] (Figures 2A,B). Both purple and blue lights were administered at photopic light intensities of 1.2 × 10^13^ photons/cm^2^/s (13.1 log quanta/cm^2^/s), while 5 x 10^13^ photons/cm^2^/s (13.7 log quanta/cm^2^/s) was selected for red light administration (Figure 2A). The photon densities for each light stimulus were chosen based on the previously determined irradiance response curves to monochromatic light for melatonin suppression (8, 9). In addition, since bistability was demonstrated in human PLR experiments using a higher irradiance of red light (32), a similar decision was made for the current study.

**Figure 3:**
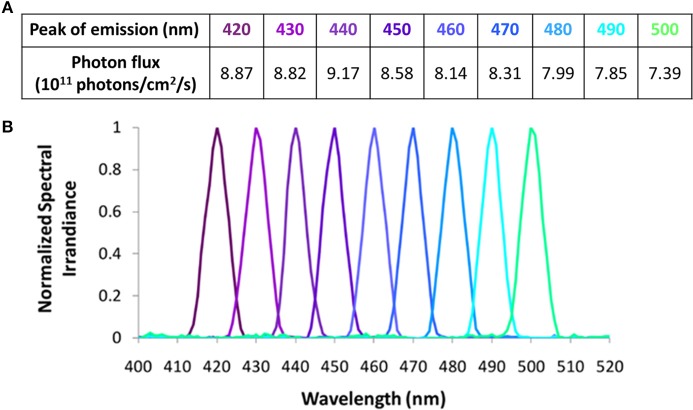
Light wavelengths tested in Study B and their corresponding photon fluxes **(A)**, and normalized spectra **(B)**.

In Study B, 9 × 10 nm increment monochromatic lights (420, 430, 440, 450, 460, 470, 480, 490, and 500 nm), each with a half maximal bandwidth of 7 nm, were obtained using a Bentham M300 monochromator (Figures 3A,B). Technical characteristics of this system have been previously described (10). Due to the narrower spectral range selected and the wavelength dependent grating response of the monochromator, the photon densities tested (Figure 3A) were 10-fold lower than the ones achieved in Study A (Figure 2A). The achieved photon density was approximately 11.9 log quanta/cm^2^/s at the level of the cornea. Considering a 0.3 correction for optical media, all the photon fluxes tested would have been applied above 11 log quanta/cm^2^/s, thus being above the photopic threshold (43) and within the limits for melanopsin activation (18). Identical photon fluxes could not be obtained for all the wavelengths tested due to technical limitations of the instrumentation. Some data from Study B have already been published as part of a previous study in which they were correlated with different aspects of the circadian system (10).

The tested range of wavelengths were selected according to previous studies on short wavelength sensitivity of the human circadian system (8, 9, 44–46). In addition, assessing the effects of wavelengths shorter than the melanopsin λ_max_ peak on the PLR could help to clarify the role of very short wavelength light.

Since two of the lights in Study A and B were almost identical in terms of maximum spectral emission (peak at 437 nm in Study A vs. 440 nm in Study B and peak at 479 nm in Study A vs. 480 nm in Study B), comparison between a higher (1.2 × 10^13^ photons/cm^2^/s) and a lower (8–9 × 10^11^ photons/cm^2^/s) photon density at those wavelengths was performed.

#### Pupil recording

To assess the consensual PLR, a pupillometer system was used. The pupil size was tracked from the infrared illuminated (left) eye through a video pupil tracking system (ViewPoint Eye Tracker^®;^, Arrington Research Inc., Scottsdale, AZ). The researcher helped the participants to be seated in front of the sphere in darkness, resting their forehead and chin on the pupillometer system support while the left eye was focussed by the infrared camera. The system recorded 220 data per second [see (10) for further technical details].

### Data analysis

#### Data pre-processing

Pupil diameter was analyzed using software specifically designed by the Chronobiology Laboratory and the Artificial Intelligence Group at the University of Murcia (Pupilabware®), as already described (10). This processing included the determination of baseline (mean pupil diameter during the 60 s in darkness prior to the light stimulus) and normalized pupil size (NPS), i.e., ratio of the measured pupil diameter divided by the baseline pupil size.

#### Primary pupil outcome parameters

**Figure 4:**
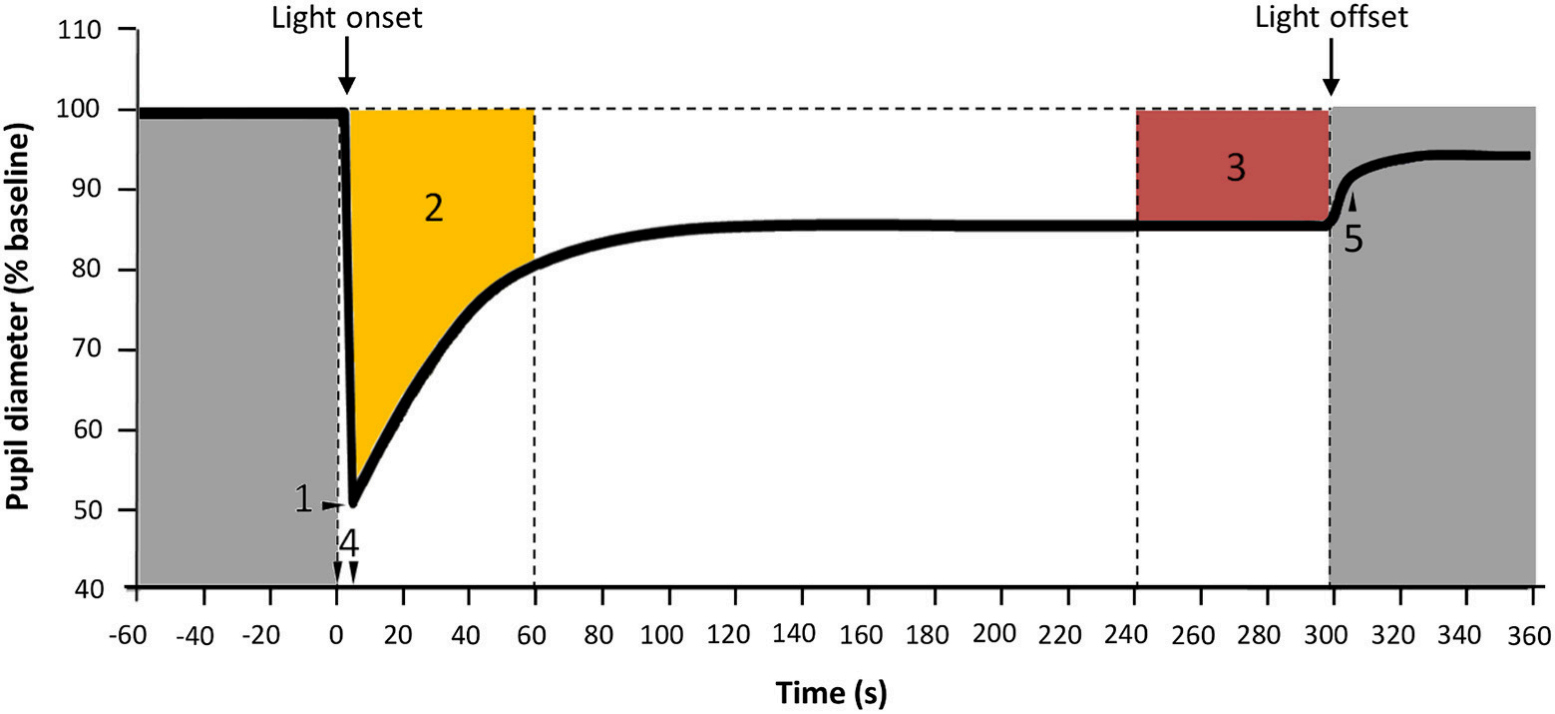
Parameters assessed for PLR. 1: Maximum constriction; 2: Area under the curve from 0 to 60 s of light exposure (AUC_0−60_, yellow); 3: Area under the curve from 240 to 300 s of light exposure (AUC_240−300_, red), expressed in A.U.; 4: Time from light onset to the minimum pupil diameter reached during constriction; 5: 6-s PIPR. Gray areas indicate lights off.

Minimum diameter (expressed as relative maximum rapid pupil constriction), time to minimum (time required to achieve the relative maximum rapid pupil constriction), velocity of pupil constriction as $\left(\frac{\text{maximum constriction}}{\text{time to minimum}}\right)$ and area under the curve ($AUC=AUC\overset{t1}{\underset{t0}{\sum }}100-NPS$), where t_0_ is the initial time point of pupil response and t_1_ is the end time, 100 is the baseline pupil size, and NPS is normalized pupil size. Two AUCs were calculated: AUC_0−60_, corresponding to the first minute of light exposure and AUC_240−300_ that corresponds to the last minute of light exposure within a 5 min light stimulus. AUC was expressed as “arbitrary units” (A.U./AU) (Figure 4). Pupil diameter 6-s after light offset (6-s PIPR) was calculated in Study B.

#### Statistical analysis

All statistical analyses were carried out using SPSS 25 (SPSS Inc., Chicago, IL, USA). When not all participants received all light conditions, missing parameters were replaced by the average parameter under that light condition. For parameters that did not have a normal distribution Friedman's non-parametric test for related samples (*post*-*hoc* Wilcoxon) was performed instead of repeated measures ANOVA (Bonferroni *post*-*hoc*). The significance level was set at *p* < 0.05. Bonferroni correction was applied after *post*-*hoc* pairwise comparisons. When only two conditions were compared, a paired or unpaired Student's *t*-test (or Mann-Whitney U) was performed. All the results were expressed as mean ± standard error of the mean (SEM).

### Photoreceptor activation

The photoreceptor activation was assessed for each light condition using the Irradiance Toolbox (v1.), developed by Lucas et al. (47), that calculates the α-opic lux parameter, which in turn represents the excitation of each of the 5 photoreceptors under different light spectra. This calculation is based on the estimated sensitivity curves for each photoreceptor (47). Both absolute values (obtained directly from the toolbox) and the relative and absolute contribution for each photoreceptor were assessed.

## Results

### Study A

**Figure 5:**
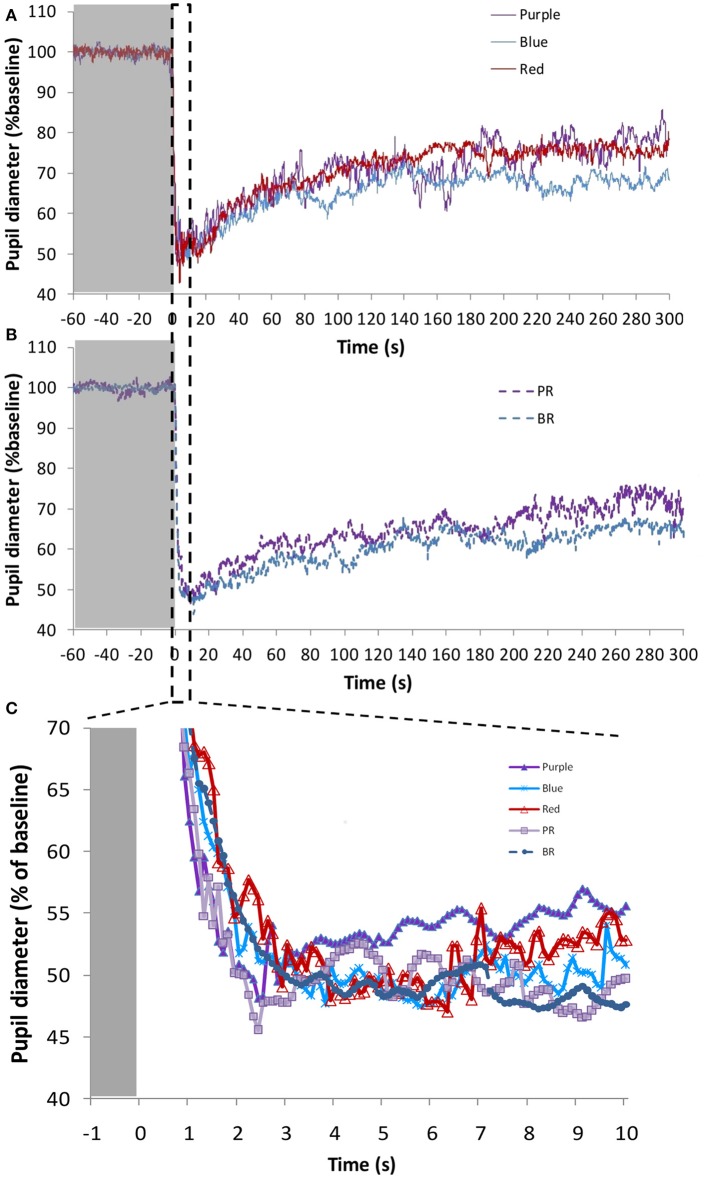
Average pupil recordings **(A)** for purple (437 nm; *n* = 3), blue (479 nm; *n* = 9), and red (627 nm; *n* = 8) light at 1.2 × 10^13^ photons/cm^2^/s (purple and blue), and 5.13 × 10^13^ photons/cm^2^/s (red), and **(B)** for purple + red (PR; *n* = 7) and blue + red (BR; *n* = 9) at 6.33 × 10^13^ photons/cm^2^/s. SEM bars have been omitted for clarity. Averaged first 10 s of pupil constriction **(C)** with error bars omitted for clarity.

Monochromatic purple (437 nm; *n* = 3), blue (479 nm; *n* = 9), and red (627 nm; *n* = 8) light stimuli and the combinations “purple + red” (PR; *n* = 7) and “blue + red” (BR; *n* = 9), were tested (photon densities indicated in Figure 2A). Figures 5A,B show the average pupil recordings for each light condition. As expected, under all tested light conditions, pupil constriction reached its minimum relative diameter within the first 10-s after light onset (transient response) (Figure 5C), re-dilating in a rapid manner during the following 50-s (escape), followed by the sustained part (steady state photoequilibrium) of the PLR.

There were no significant differences in the maximum pupil constriction following the light stimuli. Constriction under blue 479 nm light (57.5 ± 3.7%) > red 627 nm light (54.4 ± 2.3%) ≈ purple 437 nm light (54.2 ± 3.7%). Although the time needed to reach the minimum diameter tended to be shorter under the blue light condition, there were no significant differences between the different light stimuli. The velocity of pupil constriction, however, was significantly faster (one-way repeated measures ANOVA, *F* = 51.168, *df* = 2.065, *p* < 0.001) with blue light (27.9 ± 10.3%/s) than with the red (14.6 ± 1.7%/s) and purple (15.7 ± 1.1%/s) light (Bonferroni *post*-*hoc* test, *p* < 0.001).

**Figure 6:**
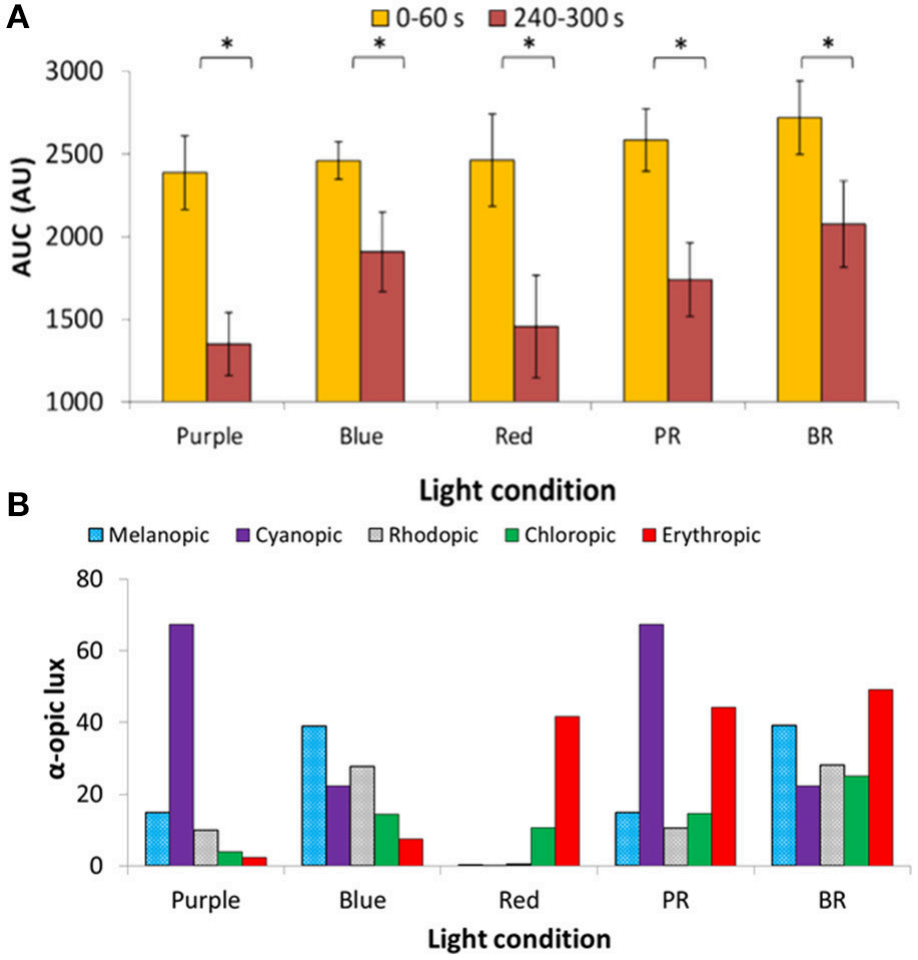
Area under the curve (AUC, expressed in A.U.) during two time windows: from 0 to 60 s of light exposure (0–60 s; first minute of light exposure, in yellow) and from 240 to 300 s of light exposure (240–300 s; last minute of 5-min light exposure, in red). Two-way repeated measures ANOVA showed an overall significant difference between 0–60 and 240–300 s (*p* < 0.05), but not between wavelengths nor interaction between both factors. Paired-sample *t*-tests revealed significant differences (indicated by *) between AUC_0−60_ and AUC_240−300_ under all the light conditions (*p* < 0.05) **(A)**. Activation of the different photoreceptors under each light condition used, assessed by the α-opic lux parameter calculated by the Irradiance toolbox (47) **(B)**. P indicates purple (437 nm; *n* = 3); B indicates blue (479 nm; *n* = 9); R indicates red (627 nm; *n* = 8); PR indicates purple + red (*n* = 7); and BR indicates blue + red (*n* = 9).

The AUC of two PLR periods (first, AUC_0−60_, and last, AUC_240−300_, minute of light exposure) was calculated (Figure 6A), to evaluate the transient and sustained response, respectively. As expected AUC_0−60_ was always higher (thus, smaller diameter during the transient response) than the AUC_240−300_ for all light conditions (Wilcoxon *post-hoc, p* < 0.017). There were no significant differences in the AUC_0−60_ (transient response) between the light conditions, with the highest value found under the blue + red light condition (2,720 ± 223 A.U.). Similarly, in the sustained response (AUC_240−300_) there were no significant differences between the different light conditions (although significant overall effect, Friedman's test, χ^2^ = 10.8, *df* = 4, *p* = 0.029), although blue light alone or in combination tended to produce a more sustained higher amplitude response (blue, 1,908 ± 241 A.U.; blue + red, 2,076 ± 260) than purple (purple 1,350 ± 191 A.U.; purple + red 1,740 ± 222 A.U.) or red (1,456 ± 311 A.U.) light wavelengths.

The retinal photoreceptor excitations were obtained for each light condition (Figure 6B) by calculating the α-opic lux parameter, a parameter which represents each photoreceptor excitation [Irradiance Toolbox (47)]. In the case of purple light, the highest activation was for S-cones (67.3 cyanopic lux, absolute value; 68.4% of the total), while for blue light, melanopic excitation was highest (39.0 melanopic lux, absolute value; 35.2% of the total). For red light, as expected, the predominant excitation corresponded to L-cones (41.8 erythropic lux, absolute value; 78.7% of the total) with less excitation of M-cones (10.7 chloropic lux, absolute value; 20.1% of the total). Rod activation was also highest under blue light, both alone (B) (27.7 rhodopic lux, absolute value; 25% of the total) and in combination with red light (BR) (28.2 rhodopic lux, absolute value; 17.2% of the total).

### Study B

**Figure 7:**
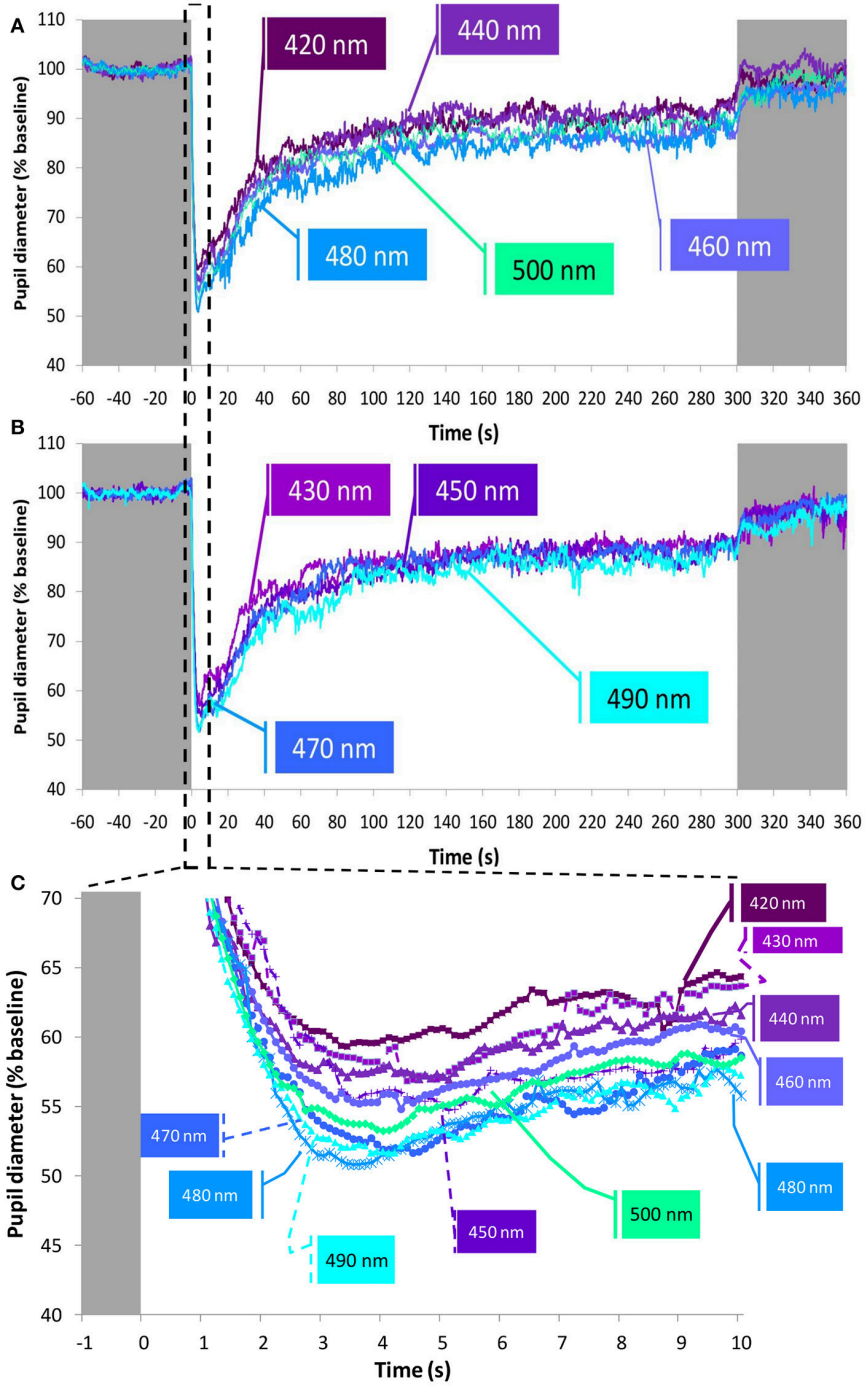
Average pupil recordings at 420 nm (*n* = 15), 440 nm (*n* = 15), 460 nm (*n* = 15), 480 nm (*n* = 13), and 500 nm (*n* = 13) light **(A)** and at 430 nm (*n* = 14), 450 nm (*n* = 15), 470 nm (*n* = 15), and 490 nm (*n* = 14) light **(B)**. SEM bars have been omitted for clarity. See Methods and Figure 3A for photon flux details of the light stimuli. Averaged first 10 s of pupil constriction **(C)** with error bars omitted for clarity.

Nine monochromatic light stimuli in 10 nm increments peaking between 420 and 500 nm were tested (peaks of emission at 420 (*n* = 15), 430 (*n* = 14), 440 (*n* = 15), 450 (*n* = 15), 460 (*n* = 15), 470 (*n* = 15), 480 (*n* = 13), 490 (*n* = 14), and 500 (*n* = 13) nm) at the photon densities indicated in the Table (Figure 3A). The average PLR for each light condition is shown in Figure 7 (for clarity, the nine wavelengths tested have been represented in two separate graphs, Figures 7A,B). As expected, typical PLR dynamics were obtained in all cases, reaching the maximum pupil constriction within the first 10-s of light exposure (Figure 7C).

The maximum relative rapid pupil constriction under the light stimuli was achieved with the longer light wavelengths (one-way repeated measures ANOVA, *F* = 5.204, *df* = 5.841, *p* < 0.001), reaching the greatest pupil constriction at 470 nm (50.2 ± 1.6%), and the smallest constriction at 430 nm (42.7 ± 1.9%). According to pairwise comparisons (Bonferroni *post*-*hoc, p* < 0.05), only the constriction at 430 nm (not at 420 nm nor 440 nm) was significantly smaller than that found at 470 nm (50.2 ± 1.6%), 480 nm (49.9 ± 1.8%), 490 nm (49.4 ± 1.8%), and 500 nm (49.7 ± 1.2 %).

It took less time to reach the minimum pupil diameter with 480 nm (3.7 ± 0.2 s) and 490 nm (3.8 ± 0.4 s) light than with 420 nm (4.6 ± 0.5 s) light, although these differences were not statistically significant. However, the velocity of constriction was different (Friedman test, χ^2^ = 29.417, *df* = 8, *p* < 0.001) with the 480 nm light stimulus eliciting the most rapid pupil constriction (14.3 ± 0.9%/s), while the slowest pupil constriction was observed with 430 nm light (9.9 ± 1.1%/s) (Wilcoxon *post*-*hoc, p* = 0.002).

**Figure 8:**
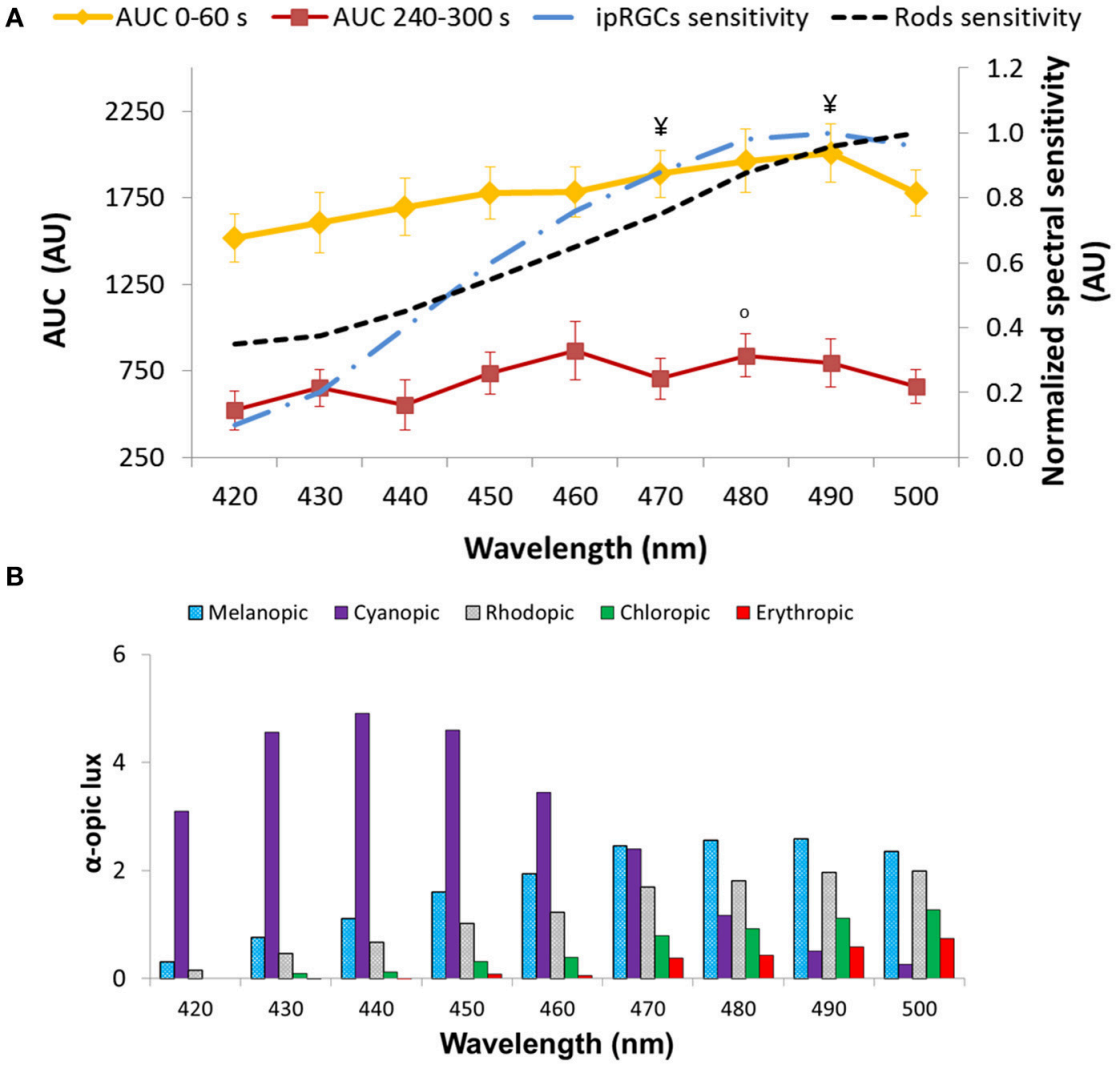
Area under the curve (AUC, expressed in A.U.) over two time windows: from 0 to 60 s of light exposure (0–60 s; first minute of light exposure, in yellow) and from 240 to 300 s of light exposure (240–300 s; last minute of light exposure, in red). Paired-sample *t*-tests revealed significant differences between AUC_0−60_ and AUC_240−300_ for all the wavelengths tested (*p* < 0.05). Two-way repeated measures ANOVA showed an overall significant difference between 0–60 and 240–300 s (*p* < 0.001), wavelength (*p* < 0.001), and a significant interaction between both factors for all light conditions. ^¥^ indicates statistically significant differences (Wilcoxon *post-hoc, p* ≤ 0.001) in AUC_0−60_ vs. 430 nm. °indicates a statistically significant differences (Wilcoxon *post-hoc, p* < 0.005) in AUC_240−300_ vs. 420 nm. Plots of the ipRGC and rod normalized sensitivities are superimposed **(A)**. Activation of the different photoreceptors according to each light tested, determined by the α-opic lux parameter calculated by the Irradiance toolbox (47) **(B)**.

Regarding the AUC (A.U.) during the transient (first minute of light exposure, AUC_0−60_), and sustained response (last minute of light exposure, AUC_240−300_) (Figure 8A), again as expected, AUC_0−60_ was always greater than AUC_240−300_ for all light conditions and wavelengths (Wilcoxon *post*-*hoc, p* = 0.001), although an interaction between both factors was evident (*p* = 0.032). Accordingly, transient and sustained responses were analyzed separately. The transient response (AUC_0−60_) tended to increase from 420 nm (1,517 ± 141 A.U.) to 490 nm (2,007 ± 147 A.U.), decreasing again at 500 nm (1,778 ± 108 A.U.) (one-way repeated measures ANOVA, *F* = 4.861, *df* = 8, *p* < 0.001). By contrast the greatest AUC_240−300_ occurred with 460 nm (880 ± 131 A.U.) and 480 nm (838 ± 100 A.U.) light (Friedman test, χ^2^ = 17.102, *df* = 8, *p* = 0.029), the latter being significantly different when compared to the 420 nm light stimulus (lowest, with 567 ± 100 A.U.) (Wilcoxon *post*-*hoc, p* = 0.005).

The 6-s PIPR could only be measured in Study B, since no recording of the post-illumination response was performed in Study A. The pupil constriction 6 s after the end of light stimulus was greater at the longer wavelengths within the 420–500 nm range (Friedman test, χ^2^ = 17.227, *df* = 8, *p* = 0.028), being maximum at 490 nm (6.82 ± 1.88%) vs. the minima at 420 (1.61 ± 1.93%) (Wilcoxon *post*-*hoc, p* = 0.001) and 440 nm (1.37 ± 1.82%).

Retinal photoreceptor activation was assessed [Irradiance Toolbox, (47)] as shown in Figure 8B. Melanopic activation was lowest when exposed to 420 nm (0.3 melanopic lux, absolute value; 8.7%), increasing at every exposure, reaching its peak at 490 nm light (2.6 melanopic lux, absolute value; 38.2%), after which it decreased again at 500 nm (2.4 melanopic lux, absolute value; 35.5%), thus showing the same pattern as the transient response (AUC_0−60_) (*R* = 0.924, *p* < 0.01). There was also a significant correlation between the sustained response (AUC_240−300_) and melanopic activation (*R* = 0.699, *p* = 0.036).

### Photon flux comparison

The effect of light intensity on the PLR was compared for purple (~440 nm) and blue (~480 nm) light considering the highest (Study A, 1.2 × 10^13^ photons/cm^2^/s or ~13 log quanta/cm^2^/s) and lowest (Study B, 8 × 10^11^ and 9.2 × 10^11^ photons/cm^2^/s, respectively or ~12 log quanta/cm^2^/s) photon fluxes.

**Figure 9:**
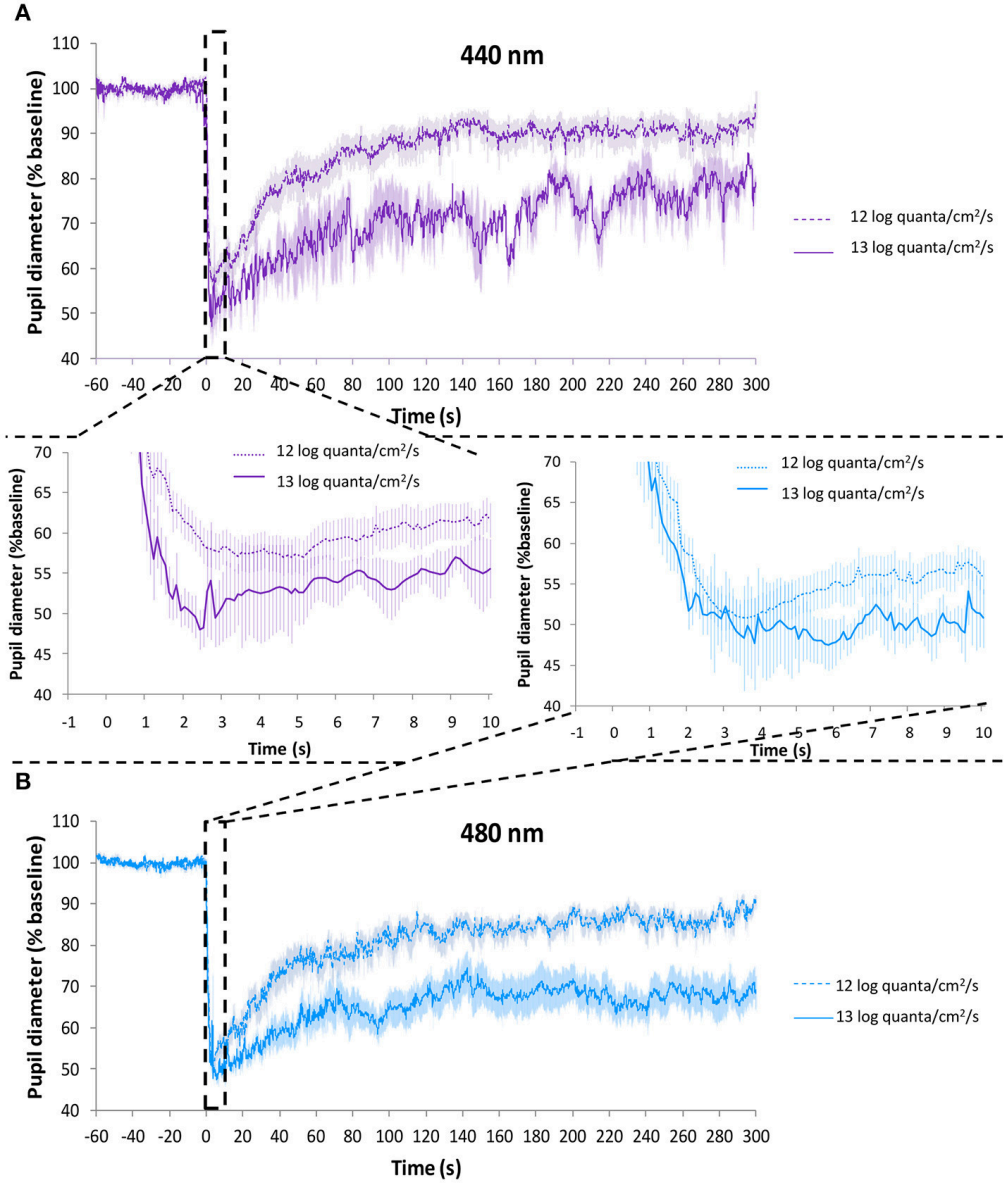
Average PLR recordings under purple light (~440 nm) at 9.2 × 10^11^ photons/cm^2^/s (~12 log quanta/cm^2^/s, discontinuous line) and 1.2 × 10^13^ photons/cm^2^/s (~13 log quanta/cm^2^/s, continuous line) **(A)**. Average PLR recordings under blue light (~480 nm) at 8.0 × 10^11^ photons/cm^2^/s (~12 log quanta/cm^2^/s, discontinuous line) and 1.2 × 10^13^ photons/cm^2^/s (~13 log quanta/cm^2^/s, continuous line) **(B)**. Error bars indicate SEM. Averaged first 10 s of pupil constriction are shown in the central panel.

Figure 9 represents the average PLR recording for each wavelength (Figure 9A, purple light; Figure 9B, blue light) at ~13 log quanta/cm^2^/s and ~12 log quanta/cm^2^/s photon fluxes. As expected, higher photon fluxes produced a greater transient and sustained pupillary constriction for both wavelengths than lower photon fluxes. The rapid pupil constriction was greater at higher compared to lower photon fluxes for both purple (54.2 ± 3.7 vs. 46.8 ± 2.3%, differences not statistically significant) and blue (57.5 ± 3.7 vs. 49.9 ± 1.8%, Mann-Whitney U test*, Z* = −2.147*, p* = 0.032) lights (Figure 9, central panel). Also as expected, it tended to take less time to reach the minimum pupil diameter under higher compared to lower photon flux for both purple (3.75 ± 0.62 vs. 4.10 ± 0.40 s) and blue (3.58 ± 1.36 vs. 3.74 ± 0.24 s) light conditions, although the differences did not reach statistical significance.

The integrative parameter “velocity of constriction” was significantly faster under higher photon fluxes for blue light (27.9 ± 10.3 higher vs. 14.3 ± 0.9%/s lower photon flux, *p* = 0.035). Purple light also tended to be faster, although statistical significance was not reached (15.7 ± 1.1 higher vs. 12.2 ± 1.4 %/s lower photon flux, *p* = 0.207). The velocity of pupil constriction was significantly affected by both wavelength (one-way repeated measures ANOVA, *F* = 5.007, *df* = 1, *p* = 0.038) and photon flux (*F* = 5.466, *df* = 1, *p* = 0.031), interaction between these factors was not significant (one-way mixed design ANOVA).

**Figure 10:**
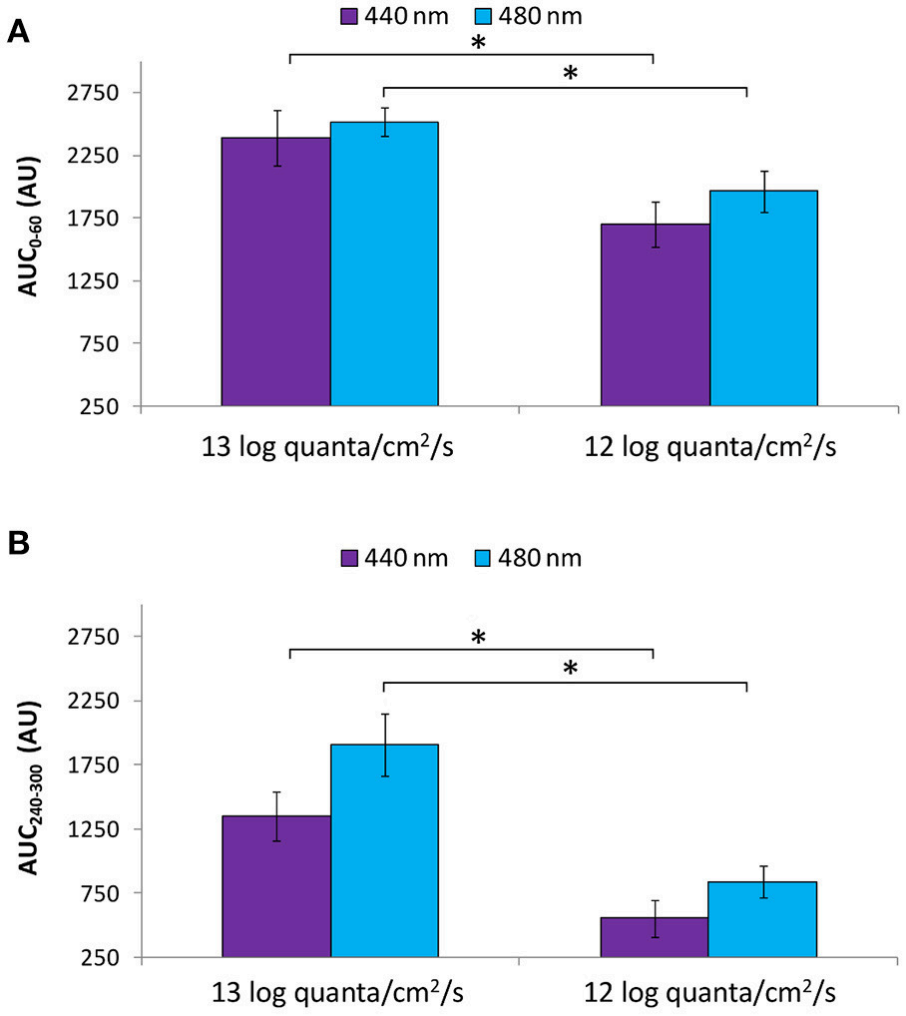
Area under the curve (AUC, expressed in A.U.) over two time windows: from 0 to 60 s of light exposure (0–60 s; first minute of light exposure) **(A)** and from 240 to 300 s of light exposure (240–300 s; last minute of light exposure) **(B)** at higher (~13 log quanta/cm^2^/s) and lower (~12 log quanta/cm^2^/s) intensity purple (440 nm) and blue (480 nm) light. Error bars indicate SEM. *statistically significant differences (Mann Whitney *U*-test, *p* < 0.016).

Both the transient (AUC_0−60_; Figure 10A) and sustained (AUC_240−300_; Figure 10B) responses were greater under higher photon fluxes for both the purple and blue light conditions (Mann-Whitney *U*-test, *Z* = −2.371, *p* < 0.016).

## Discussion

We aimed to better characterize the human PLR under high resolution, 5-min monochromatic light stimuli (10 nm increments) alone and in combination. Our results show higher responsiveness of the pupil to blue light stimuli, alone or in combination with red light, considering rapid pupil constriction, transient, sustained and post-illumination pupil responses, and velocity of pupil constriction. Higher light intensities also produced, as expected, higher responsiveness.

Traditionally when evaluating the PLR, single monochromatic lights have been tested. Some studies, however, have used combined monochromatic light stimuli (LEDs of various bandwidths) or spectrally tunable light sources, that have provided good evidence to understand the contributions and interactions of the retinal photoreceptors in the PLR (13, 15, 16, 20, 48–54), as well as regarding light-induced melatonin suppression (35). In the present study blue and purple monochromatic lights were combined with red at the same final intensity. In agreement with previous reports (32, 55) the smallest pupil diameter tended to occur in the presence of blue light (~480 nm alone or combined with red). According to the hypothesis of bistability, melanopsin may switch from a M state (not responsive to 480 nm light) to a R state (responsive to 480 nm light) by absorbing longer wavelength photons (32, 56, 57), the retinal epithelium being required for melanopsin regeneration (58). Thus, it may be plausible that red light (627 nm), when present, could elicit this conformational change, increasing the sustained response, not only due to the increased light intensity, but also to its effect on melanopsin. Although the differences did not reach statistical significance, red light tended to produce a greater effect in combination with purple light (compared to purple light alone) than with blue light (compared to blue light alone), probably because blue light can elicit the maximal intrinsic photoresponse on its own, while with purple 440 nm light, although the melanopsin is stimulated, its activation is not maximal (45, 59). This tendency, however, could also be explained by the previously described spectral opponency between S-cones and both L- cones and ipRGCs (49, 50, 54). Thus, red (activating L-cones) plus blue (activating melanopsin) light would produce a summation in the pupil constriction (13), while the combination red plus purple light would activate L- and S-cones, respectively, thus producing opponency instead of summation, resulting in smaller constriction amplitude (49, 50, 54). Other studies, however, suggest linear/non-linear summation of the 5 types of photoreceptors stimulation (51), which contradicts the opponency hypothesis. Although both light combinations were delivered at the same photon flux, the differences in pupil response due to different colors (driven by chromatic pathways) have been previously described as being 3-fold larger than those driven by luminance pathways (16).

In our study, the velocity of pupil constriction was significantly faster under blue light than under red and purple light stimuli. However, based on previous studies, the melanopsin-ipRGCs intrinsic photoresponse would produce slow and sustained pupil constrictions, while the extrinsic pathway (mainly cone-driven) would produce fast and relatively transient responses (22, 25). The findings with blue light are thus not in agreement with the expected slow response produced by the melanopsin-ipRGCs (22, 25). The delayed response previously attributed to melanopsin-ipRGCs has also been questioned in a study using a square-wave pulse, suggesting a mechanism possibly associated with cone-mediated signals (20). In addition, we could speculate that an overlapping action of S-cones may produce this more rapid response, although considering the previously described S-cone opponency, this may not be a plausible explanation (49, 50, 54).

The post-illumination pupil response (PIPR) after short light stimuli (e.g., 1-s) has been suggested as a good marker for estimating the melanopsin function (12). However, in this study we used longer duration light stimuli (5-min) in order to assess the overall PLR dynamics. Thus in order to assess the contribution of each photoreceptor we used mathematical modeling [Irradiance Toolbox (v1) application, developed by Lucas et al. (47)], which provided the theoretical photoreceptor activation under each light condition. From these results we observed that under blue light stimuli there appears to be a summation of intrinsic (melanopsin-ipRGCs) and extrinsic (rods and cones) activation, which may explain the faster pupil constriction under blue light than under red or purple light stimuli, since the latter mainly involves cone activation with little intrinsic melanopsin activation.

When evaluating the PLR under a high resolution wavelength range (namely every 10 nm) from 420 to 500 nm, the highest pupil responsiveness was observed between 470–490 nm (depending on the PLR parameter analyzed). When looking at the transient response (AUC_0−60_) a progressive increase (i.e., greater pupillary constriction) was observed from 420 to 490 nm. These PLR results are in agreement with Gooley et al. (28), who found in a blind subject with degeneration of the outer retina while the inner retina remained intact, that pupillary constriction was short-wavelength sensitive with a fitted peak sensitivity of 490 nm. The shorter (purple) wavelengths (420–440 nm) would activate S-cones so the lower amplitude pupil response found with these wavelengths may also be due to spectral opponency (49, 50, 54), these lights thus producing less constriction than the longer wavelengths with melanopsin activation. The 6-s PIPR parameter, calculated after 5-min light stimuli, also showed greater constriction (smaller diameters) with longer wavelengths, again the maximum pupil constriction being at 490 nm. 6-s PIPR has previously been found to be a good marker for melanopsin activation after 1-s light stimuli (12), so for the first time, as far as we know, this parameter has been calculated after longer light exposures. Our results also support the hypothesis that ipRGCs contribute significantly, not only to the pupillary sustained response (AUC_240−300_) (23, 28) (which tended to be greater at 460 nm), but also to the transient part of the reflex (AUC_0−60_). The transient responses showing a similar pattern to the theoretical melanopic activation further supports this idea.

The role of rods, however, cannot be excluded since rods (i) have been found to contribute to the sustained response (23) [as well as to the PIPR (24)], (ii) have a peak of sensitivity at 498 nm (60), and (iii) are partially activated under 480 nm light (as presented in Figure 8B). Cones, however, according to previous studies, contribute little to the sustained response (23, 28), since they quickly adapt to long duration light stimuli.

The PLR has been widely shown to increase with higher light intensities (18, 61, 62). In our studies (A and B) the light stimuli were similar, allowing us to compare very close wavelengths at different photon fluxes (13.08 vs. 11.93 log quanta/cm^2^/s from Study A and B, respectively). Thus, at ~440 and ~480 nm, as expected, higher photon fluxes produced a greater sustained pupillary response at both wavelengths and faster velocity of pupil constriction. Overall these results are in accordance with a higher contribution of ipRGCs at higher photon fluxes (since their activation threshold is higher) and at longer duration light stimuli (23), while cone photoreceptors would contribute to non-visual light responses at the beginning of light exposure (63).

Although pupillometry has become a useful tool to evaluate non-visual light responses, it is not problem free. The PLR has been shown to depend on wake and circadian phase (64). Thus, experiments need to be controlled for time of day, wake up time and circadian phase of the participants. In the present study performing experiments during the morning at the same clock time for each participant, as well as controlling the sleep/wake cycle of the participants 7 days prior to the PLR assessments, was designed to minimize the differences between different days, time of day and wake status. PLR could also be influenced by other processes such as changes in accommodation states, in the state of arousal or even cognitive activity (23), thus even when participants are instructed to refrain from alcohol, caffeinated drinks, bright lights, excessive exercise, and non-steroidal anti-inflammatory drug intake as in the present study, there may be additional confounding factors. Despite these limitations, a close association between the observed pupillary responses and the melatonin suppression response with monochromatic lights has been reported (9, 35, 41, 46). In addition, a relationship between circadian status and PLR has been recently proposed (10). Overall this suggests that pupillometry may, in future with more evidence from different approaches, become a practical tool to evaluate the efficiency of light sources on circadian system activation in a quick, non-invasive, and relatively inexpensive way [reviewed in (65)].

Thus, if we consider pupillometry as a proxy to evaluate circadian effects of light and considering that monochromatic blue light is most effective at suppressing melatonin (8, 9, 44), we propose that substitution of blue light by purple light in polychromatic light sources may be a solution for nocturnal illumination to minimize non-visual light responses. In order to test this it will be necessary to determine, not only whether the color discrimination is acceptable under these spectra, but also the specific effects of purple light on melatonin synthesis, sleep and alertness in humans, not only isolated, (9, 46) or in combination with blue (66) or red (35) light, but also by replacing blue light within more complex light spectra (67). In this sense, further studies about the potential risks of using purple light at the intensities required should also be conducted.

## Author contributions

MB-C, KH, SS, VR, JM, MR, and DS conception and design of the study. MB-C, KH, CI, and VR data acquisition. MB-C, VR, JM, and MR data analysis. MB-C, JM, MR, and DS wrote first draft of manuscript. All authors contributed to manuscript revision, read and approved the submitted version.

### Conflict of interest statement

VR is a scientific advisor to Lumie. The remaining authors declare that the research was conducted in the absence of any commercial or financial relationships that could be construed as a potential conflict of interest.
